# How European Fans in Training (EuroFIT), a lifestyle change program for men delivered in football clubs, achieved its effect: a mixed methods process evaluation embedded in a randomised controlled trial

**DOI:** 10.1186/s12889-023-15419-y

**Published:** 2023-03-20

**Authors:** Christopher Bunn, Victoria Palmer, Nai Rui Chng, Eivind Andersen, Cindy M. Gray, Kate Hunt, Judith G. M. Jelsma, Heather Morgan, Maria Nijhuis-van der Sanden, Hugo V. Pereira, Matthew Philpott, Glyn C. Roberts, John Rooksby, Øystein B. Røynesdal, Marlene N. Silva, Marit Sørensen, Pedro J. Teixeira, Theo van Achterberg, Irene van de Glind, Willem van Mechelen, Femke van Nassau, Hidde P. van der Ploeg, Sally Wyke

**Affiliations:** 1 School of Health and Wellbeing, College of Social Sciences, University of Glasgow, Glasgow, UK; 2 Institute for Sport and Social Science, Norwegian School of Sport Science, Oslo, Norway; 3 Institute for Social Marketing and Health, Faculty of Health and Sports Sciences, University of Stirling, Scotland, UK; 4 Amsterdam Public Health Research Institute, Health Behaviors & Chronic Diseases, Amsterdam, The Netherlands; 5 Institute of Applied Health Sciences, University of Aberdeen, Scotland, UK; 6 Radboud Institute for Health Sciences, Radboud University Medical Center, IQ Healthcare, Nijmegen, The Netherlands; 7 CIDEFES – Centro de Investigação em Desporto, Faculdade de Educação Física e Desporto da Universidade Lusófona, Educação Física, Exercício e Saúde, Lisbon, Portugal; 8 European Healthy Stadia Network CIC Ltd, Liverpool, UK; 9 Computer and Information Sciences, Northumbria University, Newcastle Upon Tyne, UK; 10 Department of Teacher Education, NLA University College, Bergen, Norway; 11 Direcção-Geral da Saúde, Programa Nacional Para a Promoção da Atividade Física, Lisbon, Portugal; 12 Department of Public Health and Primary Care, KU Louvain, Academic Centre for Nursing and Midwifery, Louvain, Belgium; 13 FWG, Department of Researchesearch and Development, Utrecht, The Netherlands

**Keywords:** Process Evaluation, Physical Activity, Sedentary Time, Football, Intervention, Mixed Methods

## Abstract

**Background:**

A randomised trial of European Fans in Training (EuroFIT), a 12-week healthy lifestyle program delivered in 15 professional football clubs in the Netherlands, Norway, Portugal, and the United Kingdom, successfully increased physical activity and improved diet but did not reduce sedentary time. To guide future implementation, this paper investigates how those effects were achieved. We ask: 1) how was EuroFIT implemented? 2) what were the processes through which outcomes were achieved?

**Methods:**

We analysed qualitative data implementation notes, observations of 29 of 180 weekly EuroFIT deliveries, semi-structured interviews with 16 coaches and 15 club representatives, and 30 focus group discussions with participants (15 post-program and 15 after 12 months). We descriptively analysed quantitative data on recruitment, attendance at sessions and logs of use of the technologies and survey data on the views of participants at baseline, post program and after 12 months. We used a triangulation protocol to investigate agreement between data from difference sources, organised around meeting 15 objectives within the two research questions.

**Results:**

We successfully recruited clubs, coaches and men to EuroFIT though the draw of the football club seemed stronger in the UK and Portugal. Advertising that emphasized getting fitter, club-based deliveries, and not ‘standing out’ worked and attendance and fidelity were good, so that coaches in all countries were able to deliver EuroFIT flexibly as intended. Coaches in all 15 clubs facilitated the use of behaviour change techniques and interaction between men, which together enhanced motivation. Participants found it harder to change sedentary time than physical activity and diet. Fitting changes into daily routines, planning for setbacks and recognising the personal benefit of behaviour change were important to maintain changes. Bespoke technologies were valued, but technological hitches frustrated participants.

**Conclusion:**

EuroFIT was delivered as planned by trained club coaches working flexibly in all countries. It worked as expected to attract men and support initiation and maintenance of changes in physical activity and diet but the use of bespoke, unstable, technologies was frustrating. Future deliveries should eliminate the focus on sedentary time and should use only proven technologies to support self-monitoring and social interaction.

**Trial registration:**

ISRCTN81935608, registered 16/06/2015.

**Supplementary Information:**

The online version contains supplementary material available at 10.1186/s12889-023-15419-y.

## Introduction

Low levels of physical activity and high levels spent in sedentary time are contributing to the increasing global prevalence of non-communicable diseases such as cardiovascular disease, type 2 diabetes and some cancers, [1] and innovative programs addressing both physical activity and sedentary time are urgently needed. Although global estimates suggest men report being more physically active than women [2], men engage less with traditional lifestyle change programs [3].

One approach to attracting men to lifestyle change is to offer programs in partnership with professional sports clubs, which is expected to attract men through the draw of the football club. [4–8] The first large-scale randomised controlled trial (RCT) to demonstrate the success of this approach was the Scottish Football Fans in Training (FFIT) weight management and healthy lifestyle program. [4] Delivered by trained coaches in professional football clubs, FFIT was designed to attract overweight men and enable them to lose weight through improvements in physical activity and diet [9]. FFIT was shown to be cost effective, with improvements in diet, physical activity and weight maintained to 12 months and partially maintained 42 months after baseline [10]. FFIT has been scaled-up through a single-license franchise model in over 70 UK professional football clubs, and scaled-out into football and other sporting contexts in Australia, Canada, New Zealand, England and other European countries [11].

One adaptation of FFIT was the European Fans in Training (EuroFIT) program [8]. EuroFIT extended the innovation from Scotland to other European countries but shifted the focus from body weight loss to improving physical activity and reducing sedentary behaviour (although men were encouraged to change their diet and lose weight if they wanted to [12]). A four-country pragmatic randomised controlled trial (RCT) demonstrated that EuroFIT was effective in increasing objectively measured physical activity, and improving diet, weight, wellbeing, self-esteem, vitality and biomarkers of cardiometabolic health, but not in reducing sedentary time 12 months after baseline [8].

Before recommending the widespread roll-out of EuroFIT, we wanted to investigate in more detail how it was implemented and whether it worked as we expected it to do. We thought this more detailed information could support future deliveries and add insight into how lifestyle change programs delivered in professional sports settings can work to both attract men and enable positive public health outcomes. Thus the aim of this paper is to examine the process through which EuroFIT achieved its effects. We do this by reporting a mixed-method process evaluation [13] designed to allow us to explore the multiple pathways and dynamics at work in how EuroFIT operated and achieved its outcomes [14, 15]. In line with our published protocol, we ask, 1) how was implementation achieved in football clubs and countries, and what was delivered? and 2) what were the processes through which the EuroFIT program achieved outcomes? [16] In answering these questions we pay particular attention to the mechanisms through which outcomes were achieved, thus allowing us to interrogate and evaluate the theory of change (presented as a logic model [16]) for the EuroFIT program. We also consider any differences in country context that might have been at work.

Before describing our methods, we outline the EuroFIT program and the theories underpinning it (full description of these have been published elsewhere [12, 16]).

### The EuroFIT program: an overview

**Fig. 1:**
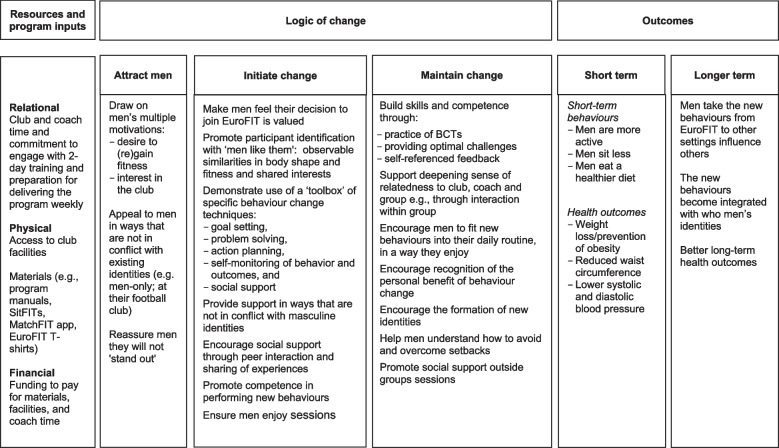
EuroFIT Theory of Change

The EuroFIT program was designed to support men to: become more physically active and less sedentary; improve their diets; and maintain these changes over the long term. It was delivered over 12, weekly, 90-min sessions that combined ‘classroom’ discussion, including ‘tools’ for behaviour change, with group-based physical activity. All sessions were led by football club community coaches. Full details of EuroFIT are published including a description of the program in the TIDieR template [16] and a summary is published in our trial report [8]. Here we describe in more detail the program theory of change (the rationale underpinning program assumptions), detailing the resources needed to deliver EuroFIT and the processes through which it was expected to work, in attracting men, and in initiating and maintaining change (Fig. 1). We also describe in more detail the theories underpinning program development and the bespoke technologies developed to support the program.

Relational, physical and financial resources are needed to implement EuroFIT, including professional football clubs with sufficient commitment to try delivering the program and to recruit coaches willing to take part in training. The program also requires access to club facilities and materials for the program, and funding to pay for materials such as manuals and t-shirts (Fig. 1). Funding for the deliveries of EuroFIT reported in this paper was provided through the research grant.

EuroFIT drew on theories derived from various schools of thought in psychology and sociology. From psychology, EuroFIT drew explicitly on contemporary theories of motivation specifically, self-determination theory [17] and achievement goal theory [18]. The goal was to facilitate participants to develop internalised, autonomous motivation that is both self-relevant and self-referenced for becoming more active, sitting less and eating a healthier diet. These theories emphasise the need to ground change in autonomous forms of motivation and to do so in a mastery-oriented, warm and supportive environment as opposed to externally imposed forms of motivation which tell men what they ‘ought’ to do. The theories also emphasise setting task-oriented, self-referenced and personally meaningful goals, rather than externally or ego-oriented goals to encourage ‘healthier living’. The change processes encouraged by these theories were operationalised in relation to initiating and maintaining change (Fig. 1) and taught the use of a set of behaviour change techniques (BCTs) [19, 20], presented to participants as a ‘toolbox’ from which they could choose the ‘tools’ they felt to be appropriate for them. These included self-monitoring, goal setting, problem solving, action planning and social support to both initiate and support change in the long term. Coaches facilitated interaction between men in sessions to help vicarious learning and to enhance motivation through enjoyment.

From sociological theory, EuroFIT – like FFIT—drew on accounts of gender, culture and performance. EuroFIT is responsive to masculine identities and practices [21], seeking to work *with* and not *against* them. For example, we expected that EuroFIT would attract men because it was male-only and based in professional football clubs, a context which is recognised as symbolically valuable to many men (Fig. 1). By avoiding prescriptive content and offering science-led options and tools for implementing them; and by delivering the program in an interactive format that encouraged mutual learning, sociability and enjoyment [22], we expected men to feel valued and challenged in a way that was congruent with their masculine identity to support them to both initiate and maintain change. We also expected EuroFIT to provide an energising environment in which participants were free to construct and rehearse new performances of the self, supported by their peers [23].

We developed bespoke technologies to support the delivery of EuroFIT. Participants were provided with a novel pocket-worn device (the ‘SitFIT’), about the size of a matchbox, to self-monitor their daily step count (for physical activity) and time spent upright (for sedentary behaviour) [24, 25]. The SitFIT can display data from the last seven days and can be connected to computers (PC or Mac) to provide a more detailed historical record of these data through the ‘MatchFIT’ app. Alongside self-monitoring, MatchFIT’s primary purpose was to encourage between-session social support and group participation in physical activity via a team-based collective step challenge [12, 16]. Each EuroFIT group use the app to represent their football club in a weekly ‘game’ against a computer-simulated team. The groups competed to achieve a team-based average step count that exceeded the opposing team’s score, which was based on an algorithm designed to challenge the team to exceed the average they achieved in the previous week’s game. Groups were also encouraged to keep in touch with commonly used social media platforms such as WhatsApp or Facebook. All materials, including SitFIT and MatchFIT were offered in the appropriate language for each country.

For the duration of the research, the country-based research teams provided implementation support for football clubs. This included funding to deliver the program, guidance on recruiting participants, provision of detailed manuals for coaches, a two-day training program for club coaches and support for trouble shooting problems during delivery. Full details of the implementation process and support offered to clubs are already published [26].

## Methods

### Process evaluation design

The mixed methods process evaluation reported here was embedded in the EuroFIT RCT (Trial registration ISRCTN81935608, registered 16/06/2015) [8]. Following UK Medical Research Council guidance, we investigated program implementation (how EuroFIT delivery was achieved and what was actually delivered), mechanisms of impact (the processes through which EuroFIT affected outcomes) and context (the broad cultural context of the country and specific cultural context of the football club in which EuroFIT was delivered). Our published protocol details eight research methods structured around delivering 18 research objectives organised around these three domains [16].

### Changes to protocol

In conducting analyses for this paper, we made some minor changes to methods described in the protocol.

The protocol described eight methods, two of which (structured telephone questionnaire with participants opting out of the study and structured questionnaires to coaches on training and on program delivery) did not yield sufficient data to support mixed methods analysis and are not included here.

The protocol did not describe two other methods which did prove useful in analysis. First, in recruiting football clubs and supporting clubs in their initial set up of the program, research teams in each country wrote implementation notes that were discussed at weekly team meetings. Both notes and discussion were useful in examining club recruitment. Second, to recruit participants to EuroFIT, clubs used social and other media adverts with a link to a web-based form for potential participants to express their interest in taking part. Data from this form were useful in examining participant recruitment.

The protocol detailed 18 objectives, but we address only 15 in this paper. We have already reported participant demographic and health risk profile, [8] and characteristics of clubs taking part in EuroFIT [27]. Club level barriers and facilitating factors are described in another paper currently under review.

### Data collection

Table 1 shows the eight methods used, organised around delivering the 15 objectives addressed in this paper, which in turn, were organised around the research questions. It also notes which aspect of the EuroFIT Theory of Change (resource, attract men, initiate change, maintain change) the mixed methods analysis can shed light on.

**Table 1:** Research objectives, Theory of Change element considered and data collection methods for the EuroFIT process evaluation

	**Study team implementation notes**	**Expressions of interest and recruitment via a web-based form**	**Baseline, post program and 12 M questionnaires EuroFIT participants** **(*n* = 500)**	**Participant attendance sheets for each session (*n* = 360)**	**Coach questionnaires: post training and post program** **(*n* = 30)**	**Participants’ SitFIT and MatchFIT usage logs**	**Observation of sessions (*n* = 30)**	**Interviews with club representatives (*n* = 15) and coaches (*n* = 15) plus interviews with club representatives of interested clubs not able to take part**	**Post program and 12 M focus group discussions with EuroFIT participants (*n* = 30)**	**Convergence** **Agreement (A), Partial Agreement (PA), Silence (S), Dissonance (D), Not Applicable (N/A)**
Q1. How was implementation achieved in football clubs and countries and what was delivered?										
1. Sources and procedures for **recruitment of clubs** and reported decision making in clubs in relation to participating in EuroFIT	X							X		A
* Element of theory of change considered—resources*										
2. Sources and procedures for **recruitment of coaches** to deliver the EuroFIT program in participating clubs	X									NA
* Element of theory of change considered—resources*										
3. Experiences of **coach training** and its usefulness in program delivery								X		NA
* Element of theory of change considered—resources*										
4. Sources and procedures for **recruitment of participants** – how men were attracted		X								NA
* Element of theory of change considered—attract men*										
5. Participation in the EuroFIT program including **number of sessions attended** and the reported **extent to which SitFIT and MatchFIT were used**			X	X		X				PA
* Element of theory of change considered—initiate change*										
6. **The number of sessions and key elements** of the EuroFIT program that were delivered by coaches							X			NA
* Element of theory of change considered—initiate and maintain change*										
7. The extent to which **coaches delivered the EuroFIT program** according to the coach manual and training							X	X		A
* Element of theory of change considered—initiate and maintain change*										
Q2. What were the processes through which the EuroFIT program affected outcomes?										
8. Participants’ reported **reasons for joining, or continuing with** the EuroFIT program			X						X	A, D in relation to 'men like me' and 'club importance'
* Element of theory of change considered—attract men*										
9. **Interaction between men** and between men and coaches during the program							X			NA
* Element of theory of change considered—attract men and initiate change*										
10. How coaches used the **coach manual and associated materials** to deliver the EuroFIT program							X	X		PA
* Element of theory of change considered—initiate and maintain change*										
11. **Coaches’ views and experiences** of the EuroFIT program and materials and in particular, which elements of the program were viewed as helpful and unhelpful in supporting participants to make lifestyle changes								X		NA
* Element of theory of change considered—initiate and maintain change*										
12. **Participants’ views and experiences** of the EuroFIT program and materials, which elements of the program were viewed as helpful and unhelpful in supporting them to make changes and how the environment coaches created influenced participant responses. Pay particular attention to the use of the toolbox of behaviour change techniques including SitFIT, MatchFIT, goal setting and self-monitoring			X						X	PA, D in relation to objectively measured sedentary time
* Element of theory of change considered—maintain change*										
13. **Participants’ experiences of maintaining (or not) any lifestyle changes** made as a result of the program 12 months after baseline									X	NA
* Element of theory of change considered—initiate and maintain change*										
14. Participants’ views of which **aspects of the program that were helpful** and which less so for supporting long-term change			X						X	A
* Element of theory of change considered—initiate and maintain change*										
15. The characteristics of coaches that delivered EuroFIT in relation to background, demographic characteristics, skills, and experiences					X			X		NA
* Element of theory of change considered—resources*										

### Research team implementation notes

Throughout the recruitment and delivery phases, the EuroFIT research team kept detailed notes on implementation. These notes were recorded in the minutes of regular meetings and provided data on issues that were faced.

### Expressions of interest and* recruitment*

Expressions of interest were captured via a web form, which provided data on the numbers of men interested in participating in EuroFIT. Potential participants were screened by telephone for eligibility from this database before recruitment began, as described elsewhere. [8, 12]

### Participant questionnaires

All 1113 RCT participants (560 allocated to intervention and 554 to comparison group) were asked to complete self-report questionnaires at baseline, when the intervention group had finished the program, (post-program) and 12 months after it began (12-months). The intervention group participants were also asked to complete additional self-report questions about their experiences of the program both post-program and at 12-months. The pre-program questionnaire asked about motivations for joining EuroFIT. Post-program questionnaires asked which components and tools of the EuroFIT program men received, used or did not use, and which they found useful. The 12-month questionnaire was designed to assess participants’ views on the ongoing usefulness of the components and tools in EuroFIT.

### Participant attendance

Club coaches were asked to keep a record of attendance at each of the 12 EuroFIT sessions, via an online database provided by the research team.

### Participants’ SitFIT and MatchFIT usage logs

Participants registered on the MatchFIT app and then SitFIT and MatchFIT usage data were collected remotely from users on a dedicated server. Data generated included logs of data uploads, error reports, user clicks, logins and logouts. Each item of logged data included a timestamp, information about the web browser and device type being used, and (except for pre-registration and pre-login activities in MatchFIT) a unique SitFIT identifier.

### Observation of sessions

Observations of EuroFIT sessions were conducted in each of the 15 clubs by members of the local research teams, aiming for two sessions per club. We aimed to observe session 4, which covers multiple topics and activities, and one of session 3 or 5–12 in each club. We avoided sessions 1 and 2 to allow groups time to ‘form’ without feelings of being studies. Data were written down in note form using a standardised proforma, and written up in the style of thick descriptions [28, 29]. They focused on how participants interacted with one another and with coaches who led sessions, how they responded to different elements of the program and any other features of the interaction which may have influenced program effects. Notes of session 4 were also used to assess whether key components of the session were delivered as intended in a measure of fidelity.

### Semi-structured interviews with coaches and football club representatives

We conducted semi-structured interviews with 16 coaches who delivered EuroFIT and with 15 football club representatives involved in managing EuroFIT when the program had been delivered.

Interviews covered what they thought of the program, their experiences of what worked or did not work when launching EuroFIT, barriers to and facilitators of implementation, whether or not they saw a future for the program in the club, and what would be needed in the future to enable EuroFIT to continue at their club. Interviews were conducted by trained members of the research team, and were audio recoded and transcribed verbatim.

### Focus group discussions with participants

Focus groups were conducted in each football club with a sample of participants who attended six or more EuroFIT sessions at two time points, when the program ended and 12 months after the program started. We tried to recruit the same men at each time point. We prompted discussion on what men thought of the EuroFIT program sessions, any perceived impacts on their lives, which elements of the program they found helpful or unhelpful and ways in which the program might be improved. Focus groups were conducted by trained moderators who were assisted by note takers, and were audio recorded and transcribed verbatim.

### Data analysis

Our approach to data analysis is described in detail in our protocol paper [16].

Quantitative data were summarised using SPSS (v21) and reported descriptively. Qualitative data (study team notes, observations, interviews, focus groups) were analysed following a framework approach [16, 30], which included the development and testing of a thematic framework through on-going discussion in the research team. The thematic framework was applied separately by researchers in each country in local languages and supported by Nvivo 11, MAXQDA and AtlasTi (depending on site). Data from each of the four sites were then summarised by theme in English by local research teams, with example data extracts translated into English. These summaries were then compared systematically using framework approach matrices by three researchers (CB, NRC and VJP) based on the research questions and research objectives. Qualitative analysts from across the four research teams discussed data extensively and checked interpretations in multiple online and offline fora throughout the analysis process.

Finally, to compare findings from different data sources, we were guided by the mixed methods ‘triangulation protocol’ [31], assessing agreement and dissonance across the datasets and also to identify areas of ‘silence’ i.e. where a given dataset has nothing to contribute, summarised in a ‘convergence matrix’ organised by objective. This final stage allowed us to examine the extent to which the data confirmed, were ambivalent toward or contradicted the causal assumptions in the Theory of change as well as any potential differences in delivery between countries.

### Informed consent and ethical approvals

When participants agreed to join the study, they completed and signed an informed consent form, in the presence of a trained researcher. Participants received information sheets explaining the study, its aims and procedures, and were encouraged to read and ask any questions they had. They were asked to provide consent for each type of data collection they were involved in. All participants gave their written consent for the use of de-identified data collected during the study to be used in publications and outputs.

Ethical approvals for the RCT and the process evaluation were obtained from appropriate country-specific ethics committees (Ethics Committee of the VU University Medical Center [2015.184]; Regional Committees for Medical and Health Research Ethics, Norway [2015/1862]; Ethics Council of the Faculty of Human Kinetics, University of Lisbon [CEFMH 36/2015]; and Ethics Committee at the University of Glasgow College of Medicine, Veterinary and Life Sciences [200140174]). All methods were deployed in this study were performed in accordance with relevant guidelines and regulations.

## Results

A summary of response rates by method can be found in Table 2. We present results in relation to the research objectives, organised around the two research questions: 1) how was implementation achieved in football clubs and countries, and what was delivered? 2) what were the processes through which the EuroFIT program affected outcomes? In each section, we also report on whether aspects of the theory of change were supported and any differences between countries which might be indicative of country-specific contexts to affect outcomes and the final column of Table 1 shows results of data triangulation in relation to agreement, dissonance and areas of silence across the datasets in relation to each objective.

**Table 2:** Response rates for survey and qualitative methods

Country/ Method*	Baseline Survey (study pop)	Post-Program Survey (study pop)	12 Month Survey (study pop)	Observations (total delivered)	Coach Interviews (N sites)	Club representatives (N sites)	Post-Program FGD** n (N sites)	12 Month FGD** n (N sites)	Participant attendance logs
NL	275 (280)	249 (280)	240 (280)	8 (48)	5 (4)	5 (4)	28 (4)	29 (4)	96 (96)
NO	255 (260)	200 (260)	174 (260)	6 (36)	3 (3)	3 (3)	18 (3)	20 (3)	71 (72)
PO	232 (233)	194 (233)	201 (233)	6 (36)	3 (3)	3 (3)	24 (3)	21 (3)	72 (72)
UK	334 (340)	245 (340)	253 (340)	9 (60)	5 (5)	4 (5)	36 (5)	37 (5)	117 (120)
Total	1096 (1113)	921 (1113)	908 (1113)	29 (180)	16 (15)	15 (15)	106 (15)	107 (15)	356 (360)

*NL The Netherlands, *NO* Norway, *PO* Portugal, *UK* United Kingdom

**FGD Focus Group Discussions

Qualitative data extracts are labelled to indicate participant ID (P (participant); C (coach); and (club representative)), club (NOR1-3, UK1-5, POR1-3, NL1-4) and data source (PPFGD [post-program focus group], 12MFGD [12-month focus group], OBS [observations], INT [interview]. Additional examples from the qualitative data can be found in Additional File 1, which presents qualitative extracts in relation to the theory of change in more detail.

### How was implementation achieved, what was delivered and by whom?

*Recruitment of clubs and coaches and experiences of coach training (objective 1, 2, 3 and 15 Table *1; element of* theory of change—resources).*

Using research team members’ professional networks, we successfully recruited 15 clubs from four countries to deliver the EuroFIT program. Study implementation notes recorded that three clubs pulled out between initial approach and commencement of training, one in Norway and two in the Netherlands. Despite research funding paying for delivery, financial difficulties and changes in club priorities regarding community engagement as reasons for withdrawal. Using research team members’ networks, it proved possible to replace these clubs within the study timeline.

Analysis of study implementation notes and interviews showed agreement that the clubs co-operated well with the research team throughout the study. All 15 participating clubs sent at least two coaches for two-days of training to deliver EuroFIT program, which was conducted in each country by members of the research team who had been involved in the development of the program. Most of the coaches were male (21/30, 70%) and aged between 19 and 56. Most were experienced football coaches, and some had additional health and fitness qualifications. In Norway and some clubs in the Netherlands some coaches were not club staff but were employed specifically to deliver EuroFIT on a sessional basis.

Analysis of interview data showed that coaches were generally positive about the training they received and felt it prepared them to use the tools they needed to be able to deliver the EuroFIT program. For example, one said:

“I liked the fact that we received a lot of relevant information. It gave me the confidence I needed. I wouldn’t have been the same with a manual only. I liked having the information up front, so I felt I knew what I was saying. It was good. Was positive. A good combination of theory and practice.” *[C-NL1-INT]*

However, analysis of study implementation notes showed a high turnover of coaching staff at some clubs in the UK with some of the coaches originally trained to deliver to program leaving their posts before delivery or part-way through the program. As a result, the research team had to develop bespoke interim training (which took one instead of two days) for new UK-based coaches.

In relation to the theory of change, the relational resources described as necessary for program delivery, were available in all countries, as were the financial resources (because they were covered by the research grant). We recruited clubs through professional networks, although additional efforts were needed to replace clubs that dropped out of the study in the Netherlands and Norway.

Attracting men to the* program (objective 4, Table *1; element of* theory of change – attract men).*

To recruit participants, football clubs e-mailed invitations to fans and used club websites, social media posts, features in the local press, and match-day leafleting/announcements to advertise the program [8]. Recruitment materials emphasised that the program took place in the football club, asking, “*Do you want to become more active with [club name]?* “and explained that “*EuroFIT aims to help you increase your physical activity levels and to be less sedentary. The program* *will offer you a toolbox of skills and techniques for living a healthy life and a chance to get fitter and feel better in yourself*.” Materials also highlighted that the program would be interactive, led by club coaches, and that they would be with like-minded people, saying; “*Each session includes physical activity led by [CLUB] coaches at [YOUR STADIUM/TRAINING GROUND] as well as focusing on how to incorporate new skills and techniques into your everyday life. The sessions will also offer you the opportunity to meet like-minded people and share tips and advice*.”

Data from expressions of interest forms showed that advertisements proved attractive to men wanting to join the program particularly in the UK and Portugal. The mean number of men per club expressing an interest in joining was 200.2 (155–272) in the UK, 121.0 (87–187) in the Netherlands, 155.3 (128–205) in Norway, and 441.0 (333–616) in Portugal. In every club, expressions of interest exceeded the number of places available (*N* = 80), with the Portuguese clubs attracting particularly high levels of interest.

In relation to the theory of change, the approach to attracting men which emphasized gaining fitness, being based in the club, and reassuring men they would not ‘stand out’ – i.e. they would be with like-minded men—proved successful in all countries, but especially in Portugal and the UK.

Participation in the EuroFIT* program and the reported use of SitFIT and MatchFIT (objective 5, Table *1; element of* theory of change* –* initiate change).*

Coach-reported mean attendance at the 12 EuroFIT sessions was comparable across the four countries: 8.7 sessions (SD 3.3) out of 12 sessions in the UK, 9.8 (SD 2.5) in the Netherlands, 8.1 (SD 3.0) in Norway and 8.7 (SD 3.2) in Portugal. Analysis of post program questionnaires showed, as reported previously [8], 65.0% of participants said they used the SitFIT ‘a great deal’ (score 4 on a scale of 0–4) and only 36.8% reported they used MatchFIT ‘a great deal’. SitFIT and MatchFIT usage logs suggest that 341/560 (60.8%) successfully uploaded SitFIT data to the server (suggesting agreement with self-reported data) and 359/560 (64.1%) registered for MatchFIT.

In relation to the theory of change, we presumed that regular attendance at the program would be necessary to initiate change, and that use of SitFIT and MatchFIT would enhance engagement. The data suggest a good level of attendance and use of SitFIT as expected.

*Number and key elements of the sessions delivered and extent to which coaches delivered EuroFIT as intended (objectives 6 and 7, Table *1; element of* theory of change – initiate and maintain change).*

As reported previously, [8] fidelity of delivery was good: we observed deliveries of the fourth of 12 sessions in 14/15 clubs and in these, coaches delivered 221 of 252 (88%) key tasks. Analysis of data from interviews with coaches agreed with those from observations. Coach training had encouraged flexibility in session delivery to suit the coaches’ own styles and the needs of the different groups, whilst retaining the core elements of the program and adhering to the principles of valuing men, supporting autonomy and positive interaction. Coaches from the Netherlands, UK and Norway, in particular described how they made adaptations to suit the needs of their group and/or the facilities available at their club (particularly for physical activity). In the Netherlands and UK, coaches reported changing the physical activity sessions to involve higher intensity activities (usually football instead of walking football), as they felt men were expecting more intense physical activity from the outset. However, some coaches also described omitting content. For example:

“I did not do the thing, filling the glasses you know [part of a suggested activity to enhance visual representation of sugar sweetened beverages in a food-related session]. I looked at the group and knew they would not be interested in these glasses and discussing how many lumps of sugar would be in them, well you know. This pouring, it doesn’t fit with this group.” [C-NL1-INT]

In relation to the theory of change, we presumed that fidelity in delivery would be important to initiate and maintain change and thus to achieve outcomes. Based on the analysis we can say that the program was largely delivered as intended because most of the key elements were delivered in the observed sessions and coaches described situations in which they were adapted the content to deliver flexibly as intended.

### What were the processes through which the EuroFIT program affected outcomes?

Participants’ reported reasons for joining, continuing with or opting out of the EuroFIT program (objective* 8, Table *1; element of* theory of change – attract men).*

**Fig. 2:**
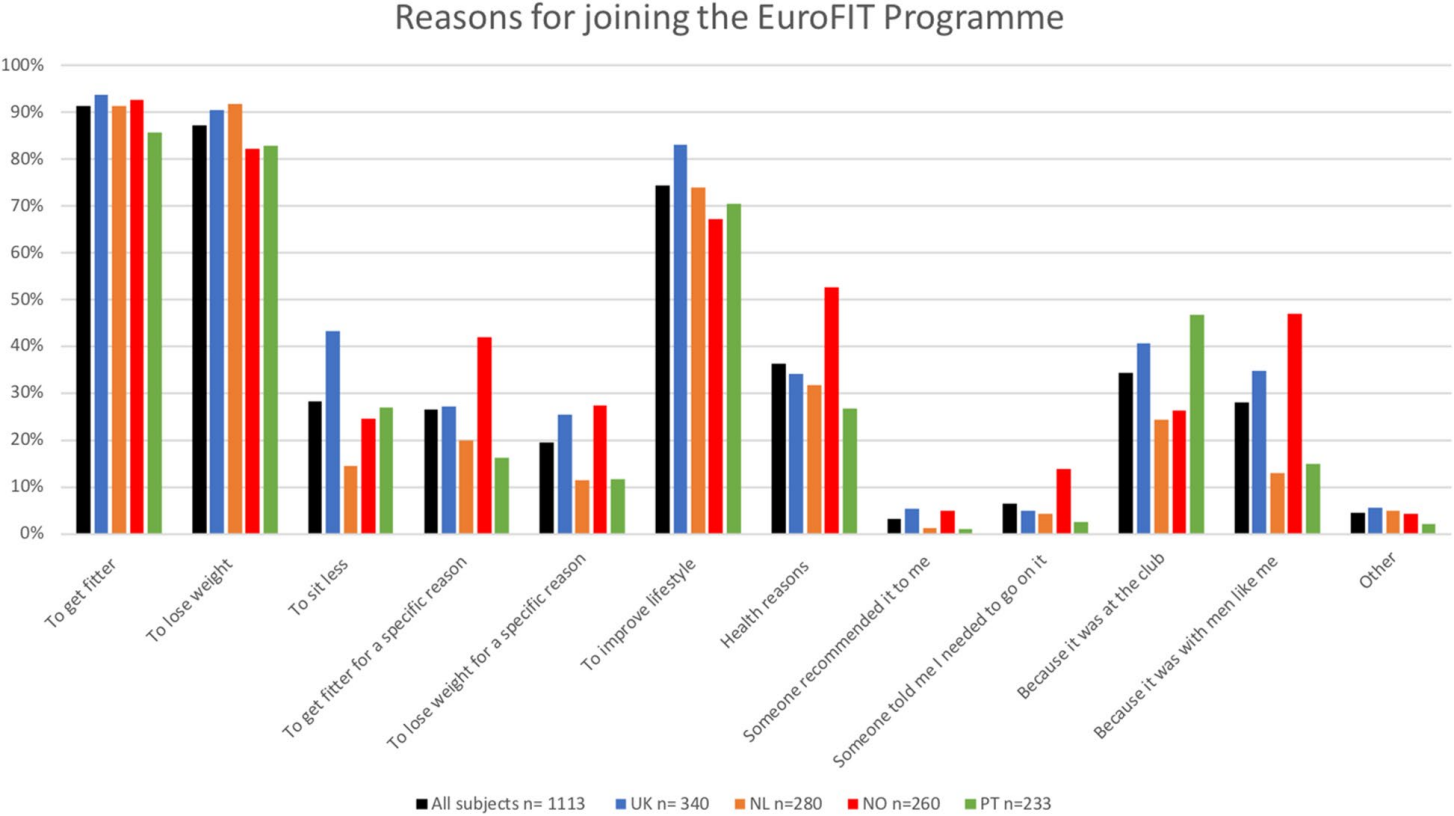
Self-reported reasons for joining EuroFIT

We have reported above that advertisements proved attractive to men wanting to join EuroFIT, particularly in the UK and Portugal. Figure 2 shows that the most cited reasons in baseline questionnaires were: to get fitter (91.3%), to lose weight (87.3%) and to improve lifestyle (74.5%). Data from focus group discussions were consistent with this. For example, one Dutch participant said:

‘So yes, I had purely looked at that, I just had something like well, there was no guarantee that you would lose weight, but you are going to exercise more, eat differently so you will get rid of some kilos. Well, that was the reason for me to start.’* [P-NL1-PPFGD]*

The importance of the club was reported as a factor by more men in the UK (40.6%) and Portugal (46.8%), than in the Netherlands (24.3%) and Norway (26.2%). This did not resonate with the post-program focus groups, which all mentioned the importance of the club as a draw. For example, in the Netherlands, one man explained how getting privileged access to valued parts of their club attracted him:

*It is still your team where you’ve been going to for years and normally you don’t get to go onto that football field. And now you are allowed to enter the field and train some more and the support you get. That’s just really important.’ [P-NL1-PPFGD]*

In the baseline questionnaires, participants in Norway (46.9%) and the UK (34.7%) were more likely to report that being with men ‘like them’ was a reason for joining than participants in the Netherlands (12.9%) and Portugal (15.0%). Again, there was some dissonance between questionnaire and focus group data, with examples of wanting to be with men ‘like them’ emerging in focus groups in the UK, Netherlands, and Portugal. For example, in a post program discussion in one UK club, participants agreed why they were attracted to EuroFIT:

P6:Meet similar people as well.

I:Yeah.

P6:The same age group, same interests and, you know, perhaps eating too many beefburgers and having too many pints [of beer], um, being able to relate and move forward together as a group. [P-UK5-PPFGD].

In the Netherlands, the importance of the program being men only was emphasised: ‘*I was thinking about that actually ….* That it was only men [–-] If there had been women I wouldn’t have joined.’ [*P-NL3-PPFGD*]. In Portugal too, one man suggested: *‘I liked the idea of being with other men of my age and condition*’ [*P-PT1-PFGD*].

In relation to the theory of change, the assumption of multiple motivations for joining the program proved correct, with men in all countries commonly citing 'weight loss', 'desire to improve lifestyle' and 'getting fitter' as reasons to join and there was agreement between data sets (see also Additional File 1). Assumptions in the theory of change about the appeal of the club were confirmed in the UK and Portugal with less evidence of its importance in the Netherlands and Norway. Similarly, assumptions about the appeal of being with others ‘like them’ were confirmed in the UK and Norway, with less evidence in the Netherlands and Portugal.

*Interaction between men, and between men and coaches,* during the* program* (objective* 9, Table *1; element of* theory of change – attract men and initiate change).*

We expected that interactions between men, and between coaches and men, would be important for group cohesion and success. The theory of change (Fig. 1) suggested that interactions with the coaches would make men feel valued and would provide support in ways consistent with masculine identities (for example, by using ‘football talk’). Session observations suggested that coaches in all four countries did encourage men to feel that their participation in EuroFIT was valued. This was made most visible through the informal ‘small talk’ (often about football) that occurred between men and coaches before the sessions began, which demonstrated a commitment to building relationships. For example, an observation in the Netherlands recorded that:

The coach then asks: “Have you guys been to the match?” “Yes,” said one of the men, “it was spectacular.” “I know right?” said the coach, “I saw you there!” “Yes, 4–3 hey?!”’ (OBS-NL1)

The way coaches valued participants was also made evident through ‘grand gestures.’ For example, in one club in the UK, a coach gave a teddy bear to a man who had just had a child. Participants in all four countries also commented on these exchanges and the welcoming nature of coaches (see Additional File 1).

The theory of change also suggested that encouraging and enabling interactions between men themselves would mean that sharing experiences would support change and that participants would enjoy the sessions and thus keep coming. In all four countries observations demonstrated that coaches encouraged interactions and men enjoyed being at the sessions. In the classroom-based sessions men shared stories of their successes, which were met with positive feedback from other men. They also reflected on more challenging experiences they had faced, when other men offered support in return:

A man who has had a visitor from [another] office tells the group of the struggle he had:

“I can’t get into my rhythm, you know, what I want to do. I have to take him to dinner and things. I’ve been good, but it’s still tougher than it should be.”

Other men sympathise and a discussion about how he might cope with such responsibilities in the future is started. Suggestions from other men include: planning the restaurants that he will take his visitor to in advance and pre-choosing his meals to avoid making impulsive decisions; tell visitors that you are eating healthily; eat carefully when you do have freedom to choose* e.g., breakfast, snacks and packed lunch; and to make sure that you have at least one nice dinner. [OBS-UK3]*

Similar supportive interactions were observed in the physical activity parts of the sessions. Although participants were often keen to do ‘better’ than other clubs in the program, there was no evidence of competition *between* men in the *same* group where interactions were supportive.

Men’s enjoyment of the program was evidenced by observations that men’s interactions were often interspersed with jokes and banter, which contributed to the supportive environment, the fostering of relatedness and enjoyment of sessions. For example, observation of a physical activity session in the Netherlands recorded:

*‘Now the men have to walk on their toes and make themselves as long as possible with their arms in the air. The string of men walk along the lines on their toes with their hands in the air. “Oh beautiful, Swan Lake!”, says the man with the bruised ribs from the sidelines.’ (OBS-NL3)*

In summary, in relation to the theory of change, the observations of sessions confirmed the assumptions we made about the importance of positive interactions in delivering outcomes (see also Additional File 1). Coaches’ interactions with men made them feel valued, and men’s supportive, humorous interactions with each other made the program both enjoyable and potentially motivational.

*Coaches’* use of the* program* manual and experience of delivering the* program (objective 10 and 11, Table *1; element of* theory of change* –* initiate and maintain change).*

Our theory of change made the coaches’ role explicit only in relation to how they interacted with men and how they encouraged interactions. Implicit to the theory of change, however, is that coaches, through following the program manual and being well prepared for sessions, would be crucial in providing an excellent motivational environment to support change [18] which included teaching participants the skills embedded in the ‘toolbox’ of BCTs.

In interviews, coaches in all countries reported that they found the manual essential for delivering the program. For example, a coach in the Netherlands said: “*The coach manual was very handy. It was really nice to have these clear instructions”* [*C-NL4-INT*].

There was agreement between the observations and interviews that coaches did teach participants how to use of the ‘toolbox’ of BCTs. For example, a coach in Norway said:

*Along with the other tools, then the SMART-goals have also been very good indeed, and those you link with the SitFIT and MatchFIT, and that is like, that you get to link those tools together has been a very good method [C-NOR2-INT*]

Many of the coaches played an important role in the repetition and practice of BCTs, and prompted men to set optimal challenges for themselves, building on their own progress, as one coach from the UK explained:

Sometimes, the guys trying to make big targets, we'd try and calm them down. Which, again, the* [training emphasised]* you need to do that, erm, which, which definitely is good advice. Erm, so yeah, I think goal setting is very important. *The guys did stick to it. [C-UK1-INT]*

However, other coach interviews showed that it could be hard to explain goal setting to participants, as one Dutch coach explained:

“I think they also found it difficult, like how to choose something [a SMART goal] that is specific enough and that I can potentially accomplish.” [C-NL2-INT]

The repetition of goal setting each week could be experienced as boring, as a coach from another Dutch club explained, suggesting that goal setting may be important in initiating but not maintaining changes:

“.. so at a certain moment, some of the men – not all of them—would go like ‘Yeah, I know by now!’ and they no longer felt like evaluating progress over and over again.” [C-NL4-INT]

Coaches clearly understood that their role was to facilitate change having created an enabling motivational environment and taught the flexible use of BCTs. This is illustrated by an extract from a coach in Norway, who said:

*I like very much that they [participants] set their own goals and that we get to choose the tools we need ourselves. We try, we give them many options to take those tools that fit into their everyday life and that we do not say that so and so and so, and if you do not do this, you fail. That you, that we make them aware, and that making your own choices is done every day, that we take those choices unconsciously in a way. [C-NOR2-INT]*

Consistent with our theory of change, analysis of observations of sessions and interviews with coaches confirmed that coaches used the manual as expected, creating an enabling environment for behaviour change by teaching BCTs.

Participants’ views and experiences of the EuroFIT* program and materials (objective 12, Table *1; element of* theory of change* –* initiate and maintain change).*

As we have seen, our theory of change placed explicit emphasis on learning to use a ‘toolbox’ of BCTs to promote an agency- and competency-based approach to initiating and maintaining changes in physical activity, sedentary and dietary behaviours. Specifically, the ‘toolbox’ focused on: goal setting, problem solving, action planning, self-monitoring of behaviour and outcomes, and social support. Data from focus groups showed that, in all four countries, men engaged with the toolbox of BCTs. For example:

*I think the great thing about the program was that it wasn't, you [didn’t have to] to make a massive amount of effort to make an improvement. It was small improvements, and you could measure them. And so, what I think was a great thing was that you learned about walking a little bit more, standing up a little bit more, just doing incremental things made you fitter. [P-UK1-PPFGD]*

**Fig. 3:**
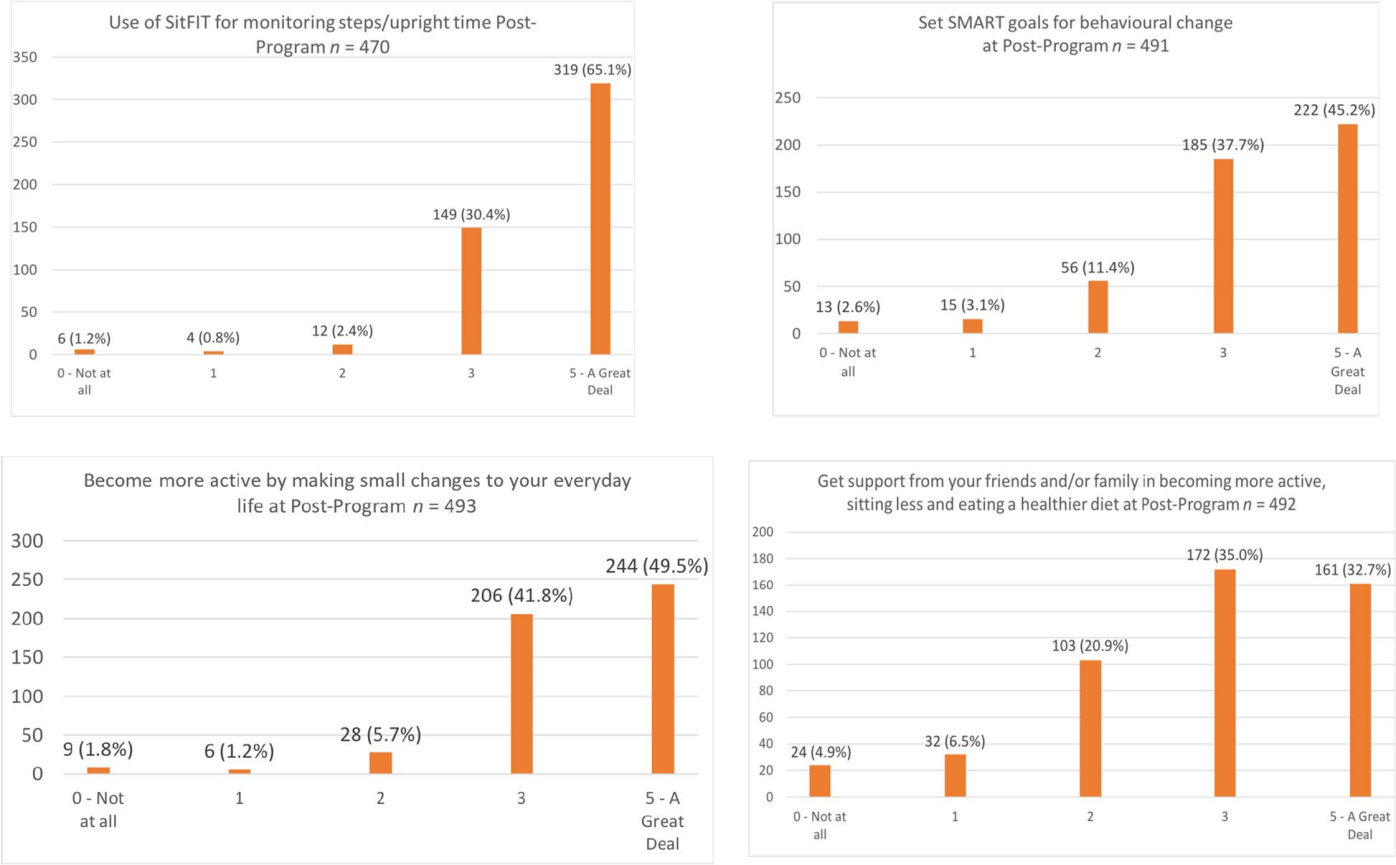
Self-reported use of BCTs at post-program

Responses to post-program questionnaires supported these findings, with participants reporting high levels of engagement with SitFIT (self-monitoring) devices, SMART goal setting, planning for making cumulative small changes to their everyday lives and seeking social support (see Fig. 3).

Objective measurements of physical activity indicated that after completing EuroFIT, on average, men were walking an additional 1208 steps/day (95% CI: 869 to 1546, *p* < 0·001) over and above the baseline average of 8,372 steps/day [8]. This finding is consistent with post-program focus group data, in which EuroFIT participants offered reoccurring narratives of being *more active*. For example, one said:

*Now I developed an excel [file] to track my routines and progress regarding my almost daily walks and runs and my weight. I really like to fulfil it and think of ways to upgrade it… I put time, intensity [into it]… look (Participant shows interviewers some printed files)’ [P -POR2-PPFGD]*

Self-monitoring was one of the main BCTs that men reported using. Men reported keeping the SitFIT device in their pockets and constantly checking their progress throughout the day or after certain activities (e.g., climbing the stairs). Across the four countries, participants commonly reported routinely using their SitFIT for monitoring their physical activity, as this interaction from a post program focus group in the UK demonstrates:

P2:I think the SitFIT is quite a big thing actually for me, you know? Err, I go out for a walk and [my wife] says have you got your SitFIT, have you got your SitFIT?

P6:Yeah, I get that.

P2:You have? Yeah, yeah, I’ve got it, I’ve got…I know I’ve got it.

P5:Or you’re gutted if you go out and you’ve forgotten it.

P3:You do, it’s the worst feeling.

P5:You can’t get those steps back.

P7:Worst feeling, yeah. [UK3-PPFGD].

As described, we developed the SitFIT (and some aspects of the MatchFIT app) to support self-monitoring. Whilst most felt positive about the SitFIT and in some cases found sharing their steps either through MatchFIT, Facebook or WhatsApp motivating, one man in the UK described how he felt that sharing his steps on MatchFIT might let the other men in his group down if he had not achieved what he had hoped (men in this group were taking a lot of steps each day):

*I think the thing as well that I noticed was I’d look at it and if it’d have, like our average would be like eighteen thousand steps, I’d look at it and go, I’m only on sixteen thousand, if I put mine, it’ll bring everybody down […]* I felt bad if I plugged it in and then…then the [MatchFIT average] figure was less after I plugged in.* [P-UK3-PPFGD]*

While men spoke positively about the SitFIT, they also described challenges using the device. These were practical (e.g., difficulty attaching device to clothing), technical (e.g., difficulty synchronising data) or mechanical (e.g., steps not registering correctly).

While self-monitoring appeared to be a useful BCT for increasing physical activity (steps), men reported that they found decreasing their sedentary time more difficult. Post-program focus group data program suggested that, while participants internalised the message to stand more, they found it harder to use self-monitoring and goal setting to change their sitting behaviour:

P5:I’ll look at it [upright time on SitFIT] and I’ll see what it is but I don’t kind of work towards it, do you know what I mean?

P2:No.

P4:It’s not…you can’t really quantify what you’re doing so…[UK3-PPFGD].

As part of the toolbox, EuroFIT aimed to encourage social support and relatedness through peer interaction. Participants responded well to encouragement to use social media for social support outside of EuroFIT sessions, using both WhatsApp and Facebook to support one another to be active, both during the program and up to 12 months afterwards (see Addditional File 1).

Another way that men were encouraged to engage with EuroFIT between sessions was through the MatchFIT app. As measured by server log data, the overall uptake (participants creating an account and using the app at least once) of MatchFIT across all countries was 63.5%. Uptake varied significantly across countries: in the UK it was 37.2%, in the Netherlands 65.2%, in Norway 78.7%, and in Portugal 82.8%. MatchFIT was introduced in week 4 of the program. About half of those who registered had stopped using the app by week 10, and, by week 12, only a third were still using it. Qualitative data from participants suggested that, while the idea of MatchFIT was appealing in all countries, particularly in the Netherlands, the practical challenges of connecting the SitFIT and uploading data limited wider engagement (see Additional File 1). Some participants also commented that playing against an algorithm, rather than other EuroFIT teams, was demotivating.

In relation to our theory of change, men’s experiences of the program, taken from post program questionnaires and focus groups, suggests that the program worked as intended for them in initiating changes to their lifestyles (particularly physical activity) and that this was consistent across countries. Self-monitoring through the SitFIT worked well, although this was less successful for sedentary behaviour than for walking and men found making changes to sedentary behaviour difficult. The motivation strategies were effective, self-referenced goal setting enabled small cumulative changes in physical activity, and relatedness through social support was important for embedding change and MatchFIT demonstrated potential to encourage interaction in between sessions but technical difficulties uploading data made its use frustrating.

Participants’ experiences of maintaining (or not) any lifestyle* changes and what was helpful in maintaining change (objectives 13 and 14, Table *1; element of* theory of change – maintaining change).*

EuroFIT was designed to support men to make changes that they would be able to sustain beyond its 12-week delivery. As noted above, practising BCTs was helpful to participants in initiating and maintaining changes through the program, and men were encouraged to make small changes that could be accommodated within their daily routines. In the 12-month questionnaire, 71% of the intervention group said they became more active by making small changes to everyday life ‘often’ or ‘a great deal’. Focus group data from all countries confirmed this, with examples such as the following offered by participants:

I’ve got to enjoy it, my routine of walking around for the* [news]paper in the morning and stuff like that, and I’ve kept it going and it’s just because it gave me the push that I needed, basically. [P-UK4-12MFGD]*

The same pattern of convergence between qualitative and quantitative data was observed in relation to the routinisation of dietary changes, in all countries. Some described such routinisation in very direct terms e.g., *“I don't make an effort… this new way of behaving is like the normal me.” [P-POR3-12MFGD].*

Our theory of change (Fig. 1) suggested that recognising the personal benefit of behaviour change would help men to maintain changes. Men in all countries reported feeling fitter and losing weight, and some had noticed energy improvements since participating in EuroFIT, often reflecting on how these changes had influenced their daily lives:

*At my home I think at least that previously when I confidently sat in the favourite chair with the remote control, then I think it is much nicer to see myself confident where I am active and that you do something with yourself to be more present at home both energy-wise and with the kids and, yeah – day-to-day stuff, really [P-NOR1-12MFGD]*

We have reported elsewhere that EuroFIT participants, on average, reduced their sedentary time post-program (i.e. at 12 week measures) by an estimated 14.4 min/day (95% CI: -25·1 to -3·8, *p* = 0·008), but that this reduction was not maintained at 12 months [8]. This finding from the RCT converged with men’s accounts in the 12-month focus groups, which described finding it difficult to integrate changes to sedentary behaviour into their daily lives. For example, one Portuguese participant said, “*Although I do some PA, changing sedentary behaviour is hard… I cannot say honestly that I accomplished it*.” [*P-POR1-12MFGD*].

As part of EuroFIT, participants were taught how to maintain new behaviours in the face of adversity, through activities which focussed on avoiding and overcoming setbacks. Participants from all four countries described how they implemented these aspects of EuroFIT, as this example from Portugal illustrates:

This weekend it was my son’s birthday, we had a big party and I had everything that I liked… Coca-Cola, beer, lots of food… I can’t even recall what exactly…Sunday I started the day by running 5 km – That’s the way to go” *[P-POR3-12MFGD]*

However, some participants expressed unhappiness about extent to which they had been able to keep changes going. For example, one man said:

“I feel guilty, I must be the worst participant ever. I think I lost about 12 pounds, but look at me now” [he had regained the weight]* [P-NL1-12MFGD]*

Such participants reported facing challenges relating to illness and injury, work patterns and culture, falling back into old habits, and life events such as divorce or bereavement. Poorer winter weather was also discussed as a de-motivating factor to continue exercise in all countries.

The theory of change also suggested that the program would nurture social support beyond the 12 weekly sessions. This was reported as happening in some clubs in Portugal, the Netherlands and the UK where groups were set up via WhatsApp or Facebook to continue to walk or play walking or five-a side football. Where this happened, it was seen as motivational was seen as important program, as in this example in the UK:

P3:I mean the whole thing when we did the EuroFIT, [..] Our group was brilliant, wasn't it? how we got on well. And it kind of continued. It's as though you handpicked them, you know what I mean? The group.

P2:I could say a few things, yes. Since we’ve stopped, actually, the program, I was playing regularly walking football. I really enjoyed that. There is such a nice group of people, I would like to call them friends, and we really were so, you know, we were getting together, playing football together, and as [name] said just recently, which was really important for me, was we were not as competitive as the others could be, so that was pure joy to play and really get physically active, and I can tell you we were really sweating a lot, you know? [UK4-12MFGD].

In Portugal, the Netherlands and the UK men who continued to do physical activity together as a group described a real strong sense of camaraderie and motivation. Men in Norway, where distances were further, did not meet up and that many of them now were walking alone (because of geographical distance and no organised sessions) and this made it hard to maintain their activity levels. They missed the group, the people and the weekly sessions they had together, and said that these things would have made it easier to maintain the activity long term. As one said ‘The mile is long if you are by yourself’ [*P-NOR3-12MFGD*].

In summary, men’s experience of the program suggests it largely worked as described by the theory of change, at least for those who maintained change – in that they recognised the personal benefits brought by relatively small, but cumulative, routinised, lifestyle changes, although not everyone had been able to keep those up. There was also evidence that men had learnt to avoid setbacks and plan for difficult encounters, although again not all participants were able to do this because of illness or other life events. Groups formed to continue exercise after the program were highly valued where they happened and were seen as motivational but, in their absence, as in Norway, it was harder to motivate ongoing physical activity.

## Discussion

Our analysis shows that EuroFIT was largely implemented as planned in professional football clubs in four countries, that coaches delivered the program as intended and that the program worked largely as expected for participants.

The research project paid for deliveries of EuroFIT within the trial context which facilitated recruitment of professional football clubs to deliver the program, although the fact that we had to identify additional clubs when three dropped out, suggests that it might be more difficult to recruit clubs if funding for deliveries was not readily available. Coaches responded well to the training provided in EuroFIT session delivery, although high turnover of staff was a problem in some clubs. Recruitment strategies proved successful in all countries, but especially Portugal and the UK and it is possible that the ‘draw’ of the football club was not so clear amongst men in the Netherlands and Norway. The UK has a long history of community development activity in professional football clubs [32], so football fans could be used to being invited to programs. Club-based activities and programs targeting adults have only recently been instigated in Portugal, although the love of football may be even stronger so the opportunity to take part in club-based activity highly motivational.

Attendance at program sessions, e assumed would be necessary to initiate change, was good. Fidelity, assumed necessary for successful outcomes, was also good; and coaches responded flexibly to participants’ perceived needs, a feature of the program we think it essential to retain in subsequent implementation [26]. Interactions between men, and between coaches and men, were also confirmed as important elements of success and as intended the environment was seen to be supportive and motivational.

EuroFIT was one of the first studies to deliberately engage motivation theory behaviour change strategies into the protocol. From both self-determination and achievement goal theories, we incorporated BCTs associated with autonomy, relatedness, competence, mastery and self-referenced goal strategies and coaches were very successful in using those strategies to foster behaviour change. Self-monitoring using the SitFIT and the setting of self-referenced goals were seen as particularly important by participants and coaches and reinforcement of BCTs was also important to maintain changes through the program. MatchFIT, on the other hand, did not seem to play an active role in encouraging maintenance of behaviour change in most countries, perhaps because of the technical difficulties encountered in its use, and unlike in the FFIT program[23] few narratives of identity change were presented by participants.

EuroFIT was not successful in supporting men to make long-term improvements to their sedentary behaviour. While there is good evidence for different approaches to, and the benefits of, becoming more active [1], less is known about how to intervene successfully to decrease sedentary time, which is often confused with physical inactivity [33]. A systematic review reported that interventions designed to increase physical activity are less successful in changing sedentary behaviour, but when interventions focus on sedentary behaviour specifically, reductions are possible [34]. Despite best efforts at the program design stage, it seems that EuroFIT may have fallen into the same trap: sedentary time did not change in the long term, and participant narratives, combined with objective outcomes, suggest that increasing physical activity (rather than reducing sedentary time) was a dominant focus for most. It is possible that the twin focus on physical activity and sedentary time limited the effort participants put into most unfamiliar of these, sedentary time. It could be that in multi-outcome focussed programs like EuroFIT different tools are needed to support changing sedentary behaviour.

That said, the mechanisms through which changes in physical activity were likely produced – i.e. goal setting, self-monitoring, action planning, social support and problem solving – are consistent with both the theory of change and with literature relating to behaviour change for physical activity in men [35]. In this way, our study adds to this body of evidence and reinforces the utility of these BCTs for interventions targeting increases in physical activity. Specifically, it confirms positive findings from other interventions targeting physical activity set in professional sports contexts [4–6, 36–38].

A novel feature of EuroFIT was its use of bespoke technologies. The SitFIT activity tracker [25] was widely appreciated by participants, but in some instances they also reported challenges in using it (e.g., practical, technical, and mechanical). These findings highlight both the potential of bespoke devices and the risk using them entails. Intervention designers considering deploying bespoke devices therefore need to consider whether the benefits offered by such devices outweigh the risk they potentially pose for intervention implementation. There are many more commercial but affordable physical activity self-monitoring devices available than when EuroFIT was developed and using one of these is probably a better option. Our findings in relation to the MatchFIT app suggest that it did not provide the platform for social support that it was intended to at least partly because of technical difficulties in uploading data. This finding is consistent with the ‘ManUp’ study [39] and with HockeyFIT [40], both of which sought to promote social support for physical activity and lifestyle change through app-based social support, which reported low levels of social engagement and rapid drop off [39].

Our study suggests that it is possible to implement EuroFIT in different club settings. It also indicates ways in which clubs tailor EuroFIT to their own contexts and characteristics. Future research and practice relating to the delivery of health interventions in professional sports settings should ensure programs are designed with flexibility in mind, to ensure that variations in resource availability do not present barriers to implementation, although the key elements of how the program functions should be maintained. Given the significant public health benefits that have been demonstrated by such interventions [4, 10, 41], and the contributions they can make to population health [42, 43], care and attention needs to be paid to the implementation contexts with the potential to maximise these benefits [44]. In addition, as we have previously suggested [26], an important requisite for future roll-out of EuroFIT would be a strong delivery partner organisation to ensure financial and human resources, while ensuring continued quality of delivery in clubs.

A final point of discussion raised by our study relates to harnessing the appeal of professional sports club settings and in being with other like them to attract participants to health interventions. As we have reported, our findings generally support the body of literature which argues that professional sports club settings are a powerful motivator for intervention participants, and perhaps particularly men [4, 5, 36, 37, 45, 46]. However, the baseline survey findings from Norway and the Netherlands suggest that it cannot be taken for granted at least for local clubs. Researchers intending to harness the appeal of professional sports organisations in new contexts should therefore consider the social and cultural forces which these organisations are subject to. In the field of football studies researchers have documented the multiple ways in which fandom manifests [47–49]. In Norway, for example, football fandom is increasingly transnational [50], and both British and Norwegian fans have polygamous relationships with different clubs [51]. Integrating interventions into localised fandoms is likely to ensure that the appeal of the professional sports-based setting is maximised and that interventions engage with these fanbases with appropriate sensitivity.

### Strengths and Limitations

A major strength of this study is that it analysed data from four countries, in fifteen club settings, and derived the findings from a large body of data collected through multiple methods, allowing for triangulation across data sources. The study also deliberately employed behaviour change strategies informed by contemporary motivation theories. These strengths and the findings from the study underpin our confidence in the utility of the EuroFIT theory of change as a representation of the program’s mechanisms of action for men in the four countries studied. A limitation is that although we went to considerable efforts to standardise qualitative data collection and analysis across countries it was conducted by multiple teams, with varying levels of experience, in four different languages. The research team which produced the convergence matrix had limited ability to go back to original sources to check interpretations for themselves. This was mitigated through extensive cross-country dialogue, but remains a shortcoming that is faced in all cross-cultural work in which all analysts are not poly-lingual (see e.g., [52]).

## Conclusions

This paper has explored the processes through which EuroFIT achieved its outcomes and the extent to which the underpinning theory of change represented the program’s mechanisms of action. Through a mixed-methods approach, we have concluded that the theory of change is an effective representation of the EuroFIT program, with only three small areas of ambiguity. In relation to this latter point, we have suggested: that the dual focus on changing physical activity *and* sedentary time may have led participants to select the behaviour they found most easy to grasp and alter (i.e. physical activity); that bespoke technologies can be complex to implement and bespoke social support platforms may have low take up; and that the appeal of the club as a site for health-related change may be culturally-mediated. Future deliveries should consider whether the distinction between decreasing sedentary behaviour and increasing physical activity is worth pursuing, should use easily available technologies to support self-monitoring and social interaction and how different forms of football fandom can be exploited to attract participants.

## Supplementary Information

**Additional file 1.**

## Data Availability

The datasets used and/or analysed during the current study are available from the corresponding author on reasonable request.
