# Supplementary material for: How European Fans in Training (EuroFIT), a lifestyle change program for men delivered in football clubs, achieved its effect: a mixed methods process evaluation embedded in a randomised controlled trial
Source: BMC Public Health. 2023 Mar 20;23:526. doi: 10.1186/s12889-023-15419-y (PMC10026416; doi:10.1186/s12889-023-15419-y)
Supplement: Supplementary file 1 — Additional file 1. [file 12889_2023_15419_MOESM1_ESM.docx]

**Additional File 1: Qualitative data extracts mapped to theory of change**

| **Theory of change category** | **Supporting data** |
| --- | --- |
| **Attracting men** |  |
| **Examples of common reasons for joining EuroFIT: weight loss, fitness and lifestyle change** | *“I've felt for a few years that I've noticed a sort of lack of fitness, and it was just that extra push I needed, just something to, to sign myself up to, to, to have that motivation, to try and get, get some level of fitness back again.”* ***[P-UK2-PPFGD]***  *“So yes, I had purely looked at that, I just had something like well, there was no guarantee that you would lose weight, but you are going to exercise more, eat differently so you will get rid of some kilos. Well, that was the reason for me to start.’* ***[P2-NL1-PPFGD]***  *‘On my part, I read that ... for us lazy supporters who were going to be fitter. I was fit once, so I thought that I might become that again. So that was the incentive for me.”* ***[P-NOR3-PPFGD]***  *“I thought it was a good opportunity to change some lifestyle pattern.”* ***[P-POR2-PP-FGD]*** |
| **Importance of the Club as a motivator for joining EuroFIT** | *“P4: I can definitely say for me it was the link to 105 City that motivated me.*  *I: Okay.*  *P4: I’d been…I mean, I’d recognised for quite a while that my life had become somewhat sedentary, and it was a case of I saw that, and I thought, well, here’s…you know, this is an opportunity for me, um, and linked it directly back to 105 City.”* ***[P-UK5-PPFGD]***  *“It might sound strange, but it is so fun to actually walk on that pitch”* ***[P-NL1-PPFGD]***  *“The reason I joined was because I was curious and because it was run by [club]”* ***(P-NOR3-PPFGD)***  *“If it was in the university, I would have said no… the true was that I only participated for my club.”* ***[P-POR2-PPFGD]*** |
| **Reflections on attending EuroFIT with ‘men like me’** | *“P4: […] Erm, the reason I came is because I'm fat. And...*  *[Laughter].*  *P5: Stocky, stocky.*  *P4: I've tried to lose weight, erm, I've tried to lose weight so many times, but my main thing is not food or diet, but exercise. It's all in my head. So, I've got no willpower...*  *I: Yeah.*  *P4: ...and I need guys like this around us, you know. And having the guys around, knowing that they're going through the same kind of crap, it just...*  *P5: [Laughing].*  *P4: ...makes you understand that you're not the only person in the world, you know, who's fat for a daft reason.”* ***[P-UK4- PPFGD]***  *“Very positive. I was thinking about that actually in the previous round. That it was only men… If there had been women, I wouldn’t have joined.”* ***[P-NL3-PPFGD]***  *“It seemed that I would be with many ‘me(s)’”* ***[P-POR1-PPFGD]*** |
| **Health Conditions** | *“And erm, because of the history of my family, where my mum and dad both had high cholesterol, and there is heart disease, I, I was always a bit concerned. I'm not young anymore.”* ***[P-UK4-PP-FGD]***  *“My cholesterol levels were over the top and I decided to do something…”* ***[P- POR1-PPFGD]*** |
| **Other reasons for joining** | “Mine was to sort of come out at the other end healthier long term so I can, um…my son’s ten and really active, so I wanted to be able to keep up with him…” **[*P-UK5-PPFGD*]**  *“…because it was in a group and I really would have never called in the help of others, so I thought I’m just going to join. Or at least I hope I’m allowed to join, and I signed up.’* ***[P-NL3-PPFGD]***  *“Yes, the message really got to me, indeed; right away. Sats (a gym) and Grethe Roede (diet plan) have similar messages, but that does not get to me, but this did” [****P-NOR3-PPFGD]***  *“I thought it was a very credible program, delivered by several partners/Universities at a European level.”* ***[P-POR1-PPFGD]***  *“I had a child in this previous year. It changed me, I thought it was time to alter my habits.”* ***[P-POR2-PPFGD]*** |
| ***Initiating Change*** |  |
| **Feeling valued** | *“P4: That encouragement, yeah.*  *P1: ...you know, they were encouraging you, or making you feel bad. And, you know, no way at all did they make anybody feel small, at all.”* ***[UK1-PPFGD]*** |
| **BCT toolkit: examples of building competence in self-monitoring and goal setting** | *“I: Okay. Erm, what about using the SitFIT?*  *P: They all got quite obsessed with it actually.*  *I: Okay.*  *P: A lot of them brought…a lot of them didn’t work though and we had a lot of issues with them.*  *I: Okay.*  *P: Erm, but they definitely did use them and some of them have continued to use them since the programme has ended. [Coach_104]*  *P: So, obviously, the SitFIT, everyone loved that, that was the main part of it.*  *I: Right.*  *P: Erm, and then, they also keep checking it, with is gonna increase their activity as well. [Coach_101]*  *P: They were, the SitFIT, they were made up with them.*  *I: Yeah. Okay.*  *P: So straight away it was like something that was brand new they had never seen it before.*  *I: Yep.*  *P: Yeah. They commented every week on oh yeah, it’s been…it buzzes in me pocket and it makes me get up and I go and do this…*  *I: Right.*  *P: …and I go and do that. They said it made a massive difference to their lives in getting active and things like that.”* ***[C-UK1-INT]***  *“They [the coaches] introduced it really nice “Oh try to take 1000 steps a day”. That is doable, you know. Everybody came back a week later [and reached their goal], except one guy who had a strange baseline week. “Well, then we will adapt yours, so you will be able to reach your goals as well”. So, after three weeks everybody reached it [their goals] and made every week small steps, I really valued that.”* ***[P-NL3-PPFGD]***  *“Along with the other tools, then the SMART-goals have also been very good indeed, and those you link with the SitFIT and MatchFIT, and that is like, that you get to link those tools together has been a very good method”* ***[C-NOR2-INT]***  *‘Now I developed an excel to track my routines and progresses regarding my almost daily walks and runs and my weight. I really like to fulfil it and think of ways to upgrade it… I put time, intensity… look (Participant shows interviewers some printed files)’* ***[P-POR2-PPFGD]*** |
| **Social support, peer interaction and ‘men like me’** | *A man who has had a visitor from his company’s London office tells the group of the struggle he had:*  *“I can’t get into my rhythm, you know, what I want to do. I have to take him to dinner and things. I’ve been good, but it’s still tougher than it should be.”*  *Other men sympathise and a discussion about how he might cope with such responsibilities in the future is started. Suggestions from other men include planning the restaurants that he will take his visitor to in advance and pre-choosing his meals to avoid making impulsive decisions; tell visitors that you are eating healthily; eat carefully when you do have freedom to choose e.g., breakfast, snacks and packed lunch; and to make sure that you have at least one nice dinner.* ***[UK3-OBS]***  *“I never forget that first night, in which we made a round around the table. There was one guy in the corner who said: in the evening I eat everything from the refrigerator. Someone else said he experienced this every night. I said I have the exact same thing. It was so recognizable among everybody.”* ***[P-NL2-12MFGD]***  *‘But the social [part] did also trigger that we managed to talk together. Because even if we are very different human beings, then we are all alike on some very elementary domains; the fact that we talk very openly about things. Everything from bad to good defecation to put it like that, after changing the diet. And that makes it so that you dare to ask some questions, and then you get some good advice’* ***[P-NOR2-PPFGD]***  *‘The group always supported and motivated me… alone it would be impossible to have done this.’* ***[P-POR1-PPFGD]*** |
| **Enjoying sessions** | *The men become particularly focussed on how to sit less. One man suggests putting a golf ball in the back trouser pocket. The room erupts into laughter, when it quietens, he smirks and says, ‘well you won’t bloody sit down, will you!’ More laughter follows.* ***[UK1-OBS]***  *The men are now in a circle. They’re doing a balance exercise. They step forward and then stand still on one leg. The men continue to make jokes. “Mister Miyagi,” says one. In pairs, they keep standing on one leg and support each other during the balance exercise. The men laugh out loud. Some pairs push each other in a teasing way and jump around together.’* ***[NL4-OBS]***  *There is generally lots of comments and laughter when the men gather in groups around the flipover boards and start to discuss. I pay most attention to two of the groups and see that some of the men are dominating the discussion a bit, which is natural. One of the men in a black shirt talks a lot in one of the groups. Lots of banter and self-irony. Midway in the discussion, one of the men shouts to the other group: "Go grab a beer". A lot of discussion underway* ***[NOR2-OBS]***  *“I would have brought cake, but the program does not allow” (Laugh)- Irony/joke of one participant in his birthday, facing the request of the others; “I am feeling so much vigour and not just here (followed by several sexual jokes)”; “Oh, XX and XX are now celebrities” (referring to participants interviewed by a national radio with regard to the program).* ***[POR1-OBS]*** |
| ***Maintaining Change*** |  |
| **Practising BCTS, optimal challenges and self-referenced feedback** | *I think my diet’s suffered slightly, I haven’t been able to maintain it as good as I should have done, but I still use a lot of the techniques that we picked up, so you only burn so many calories if you’re sitting down, so walking around, standing up a lot more, still maintain that. I’ve gone from a SitFIT to a Fitbit, because I use that to motivate me with how many steps, and this also does flights, so it emails you and it tells you, you’ve gone as high as a 747 or whatever, and you’ve climbed this mountain, so that’s motivation, and that’s all come from the course, so diet wise, not brilliant. I’ve just started walking football again, because I was running and then I’ve just been so busy, to run takes me a long time, and I just didn’t have the time, so walking, and as I say, standing and using this as a motivator to keep moving. It’s beneficial.* ***[P-UK5-12MFGD]***  *‘The coach says to the group: “the smart goals that you have made in week 7 for food and drink. That is page 75.” The men go through their workbook. One guy didn’t bring his workbook with him, but the rest of the men did. The coach proposes to look back at how it’s been going with the goals they wrote down in week 7. “Who had something with food?”’* ***[NL3-OBS]***  I did get a routine of using the weight scale. And I have not used that in 30 years one might say. And then you have a parameter there that you have some control over then. **[P-NOR-12MFGD]**  *“It was interesting, I became so addicted that I now bought a health smart watch – it tells me everything, not only steps”; “I still use it, every day, I like at night to look at it and check how things went”; “It was useful to help me raise my awareness, now I don't need the device every day… but I still use it from time to time… sometimes I say to myself… “Let’s see what it tells!”* **[*P-POR1-12MFGD*]** |
| **Fitting changes into daily routines** | *P: And also, being aware like…so just being aware maybe to sort of like exercise that little bit more. I know a couple of the guys have got into routines of they were going walking to the shops and, you know, one of them was walking on their lunch break and stuff like that. So, I think that’s,*  *I: Yeah.*  *P: …you know, once…because it was for the twelve weeks and they were keeping it up hopefully they will, you know, those habits will be ingrained.* ***[C-UK4-INT]***  *‘I’m going grocery shopping every night after dinner to get food for the next morning. And then my wife says to me sometimes: ‘Why not just get food for the whole week?’ But then I’m like, yes but then I have no purpose for walking tomorrow. I like it a lot.’* ***[P-NL1-12MFGD]***  *I did get a routine of using the weight scale. And I have not used that in 30 years one might say. And then you have a parameter there that you have some control over then.* ***[P-NOR2-12MFGD]***  *I don't need the SitFIT anymore, it is my routine… I go for a walk, and I know how many steps I got.* ***[P- POR1-12MFGD]*** |
| **Recognising benefits of change** | *I think the benefits are engrained, the way the programme’s structured, a lot of the benefits stay with you, don’t they, the food portioning, looking at the coloured labels, the sort of shock treatment when we held those weights and how much is a sugar, and things, so some of that just stays with you, it becomes the norm, so I feel equally as good as I did at the end****. [P-UK5-12MFGD]***  *“I couldn’t really do anything sports-wise when we started. And a month ago I ran the ten kilometres here in Eindhoven. In just over an hour, so it was unthinkable for me that I would have been able to do that a year ago, so to say. Uhm, throughout the year I actually did stabilise.”* ***[P-NL2-12MFGD]***  *Coach 2 is taking over and talks about benefits of becoming more active and how to measure progress. She reads right out of the manual and repeats herself several times. Then she gives the examples listed in the manual and ask they say something about which benefits they have felt. A man says that for the first time in long he can put down the eating tray on the airplanes because he has become slimmer. He seems quite pleased with this and keeps a serious face even though the men and the coaches laugh quite loud at this. Coach 2 comments, "Cool". The same man then mentions that it is easier to get up of the sofa* ***[NOR3-OBS]***  *“For me EuroFIT meant physically, mentally and social well-being.”* ***[P-POR5-12MFGD]*** |
| **Avoiding and overcoming setbacks** | *P5: I think you can have a treat. Don’t do it all the time but you’ve got to treat yourself.*  *P4: But you’re more conscious of what you’re ordering when you’re out for a meal.*  *P7: You’re more conscious of everything now, aren’t you, food-wise, sugars and everything else.*  *P6: But when that happens, you’re not particularly bothered about it because you know that you’ll go back to your normal routine, and it will come back down again. It’s just the little waves.* ***[UK2-12MFGD]***  *P5: We all fall off; it’s just about catching yourself and getting back onto it.* ***[UK1-12MFGD]***  *‘But what we do still do is fire each other up a bit, like when I see that he hasn’t done anything for two weeks and then I send everything I have done. Then I say something like: ‘Time to get out of that chair again.’ And then I get a response like: ‘Yes yes, I had an injury there for a little while, but I’m starting again, and I needed this push.’ We do need that little push at times.’* ***[P-NL2-12MFGD]***  *P3: No, it’s about planning there too. Therefore, in… I… now… as I said when autumn came, then I planned a lower activity level because I need to prioritize studies. And when Christmas came, I knew there would be some circumstances where measuring number of steps was really just to...*  *P4: Forget…*  *P3: … give a damn about, also when you go to the cabin, so if you… at least when you go with friends, and there it was… then I know… then there are a lot. So, upon till then you need to be extra good, and then I can have my reward, however I shall achieve something there to right. However, it has not been so much about food and alcohol, but it has been like even all is just lying in the sofa with a hangover I will go for a walk.* ***[NOR3-12MFGD]***  *“After the injury and because I wanted to stay active, I started to do hydro-gymnastics”* ***[P-POR1-12MFGD]*** |
| **MatchFIT** | P4: So, I've got, sort of, mixed feelings about the MatchFIT site.  I1: Okay.  P4: Because I feel it's useful to look back over previous weeks, because it keeps a log of everything that you've got from the SitFIT.  I1: Uh-huh.  P5: So, you can sort of see your improvements, and see weeks where you haven't done so well, and you can get the breakdown... **[UK2-PPFGD]**  *‘Well, there was too much hassle with signing up and registering and downloading. I don’t think that went smoothly once.’* ***[C-NL1-INT]***  *It was quite a bit of bogus with it [MatchFIT] this summer (consensus in the room) so then we could not download, and then we could not get on it at all* ***[P-NOR2-12MFGD]***  *“Although being a great concept implementation backfired… due to many technical problems which we were unable to solve participants were discouraged… making such an effort and not being recognized by it… participants were unable to upload their steps in crucial times for the team”* ***[P-POR3-PPFGD]*** |
| **Social support outside group sessions** | *P6: And I think the Face…the Facebook…*  *P7: Yeah, it’s made it…*  *P6: …much as I was against Facebook as you… know… No, but I think…I think that’s extremely powerful.*  *I: So how did you…did you…did you set up…that up yourselves or…*  *P7: Yes.*  *…*  *P3: Even when you’re not feeling like…a few lads have felt a bit down, haven’t they, and put it on there…*  *P7: Yeah, yeah.*  *P3: …and we’ve all sort of geed up and…*  *I: Yeah.*  *P3: Umm, it’s a good tool.*  *I: So that contact with one another has helped then, is what you’re saying?*  *P1: Oh yeah.*  *P5: Massive, yeah.*  *P2: Yeah.* ***[UK3-PPFGD]***  *‘But uh yeah, we’ve got 5 guys left and we have a WhatsApp group and well we app almost every week.’****[P-NL2-_12MFGD]***  *I think many are still having Facebook-contact. It’s on that level I think, contact wise. Its maybe someone, I don’t know for all… but its maybe someone having a private contact, but it seems like that Facebook-contact has been upheld by many at least…* ***[P-NOR2-12MFGD]***    *“Coaches also use the group (social media), they are always commenting on our posts, posting some tips and tricks, challenging us”* ***[P-POR1-PPFGD]*** |
| ***Relatedness to club, coach and group*** | *P3: Yeah, I think it's the chat, as well, that helps, when you're all being in the same mind, and we're all [fans of club] ...*  *P5: Yeah.*  *P3: ...and we're all sitting round with the gym equipment, chatting, and exercising at the same time.*  *I1: Yeah.*  *P3: So, it's not a chore.* ***[UK2-PPFGD]***  *‘One participant puts an arm around the coach and says to us: “This is really a great girl. We’re very fond of her.”’* ***[209_obs2]***  *‘But we did talk about that we wanted a sort of culture that it is in a football dressing room from day 1, and that the comments are flying a bit and that it should be, sort of, the [club]-culture should reflect on EuroFIT, and then it works really well. So, the talking was very free and easy, and it was, no, it was a good flow, and people looked forward to the Tuesdays and they were sad that is was over really’* ***[C1_311]***  *“They (club) treated us specially… I know they gave us a great opportunity… each week we visited different facilities… and the academy… oh the academy! … we went far beyond regular fans… it was great.”* ***[P-POR1-12MFGD]*** |
| **Forming new identities** | *“I feel just the same, I’ve lost some pounds but to say I feel better or have more self-esteem. No, I can’t say I notice anything different.”* ***[P-NL3-12MFGD]***  “Starting to see myself as a different man and I want to maintain this new image” ***[P-POR1-12MFGD]*** |
| ***Social Support*** | *“So, like, erm, I mean, l mean, for me, you know, like, erm, I had my exercise bike, so I would put on my bike in every day. And erm, to me, that was the best change that I made, 'cause I could only do, like, fifteen minutes. But then, I got up to, like, thirty minutes - ten miles in thirty minutes. And I was damn proud of that, so, of course, I put it on the days, and put it on the WhatsApp, and stuff, and it was good to be able to share with people who also were doing this stuff, and you know, you got a comment or two, and stuff like that, so that was good.”* ***[P-UK2-PPFGD]***  *“The guys helped me through, that was amazing, it meant a great deal.”* ***[P-NL2-12MFGD]*** |
| ***Short Term Outcomes*** |  |
| **More active** | *P7: The walking's made a hell of a difference to me. I'm, I was probably averaging, when we started, about six thousand paces a day.*  *I1: Uh-huh.*  *P7: I'm now averaging well over fourteen thousand...*  *I1: Yeah.*  *P7: ...on average. Some days, I can get twenty-five.* ***[P-UK2-PPFGD]***  *“I have a hybrid car. This car needs to be charged and I do not have a charging station in front of my house. I park my car a kilometre away in the next village. This is a win-win situation, my car is charged, and I have my walk.”* ***[P-NL2 -PPFGD]***  *P4: Well, I have become better at leaving the car and to walk unless it is not too far where I am going. If you are going to the store, then you walk to the store and walk to work instead of driving the car and ... That is actually pretty much what I have done [laughter].  I: Yeah, yeah, but that ...   P4: But it is like an hour, hour and a half more of walking a day* ***[NOR3-PPFGD]***  *“I walk around 1 hour each day; it is something sacred to me. If I am not able to walk in a busy day, I go for a shorter walk (30 m) after dinner”* ***[P-POR3-PPFGD]*** |
| **Sit less** | *P4: I mean, I concentrated more on the steps, to be honest, than the standing.*  *P2: Yeah, the steps.*  *P4: Because you can have the standing, the standing, I looked at, but I didn't focus that much on that.*  *P2: Were you finding when you do steps, your standing goes up as well?*  *P4: Yeah.* ***[UK1-PPFGD]***  *“I have a bar in my kitchen at home and used to sit there. Nowadays during breakfast and lunch I stand. That is about an hour standing a day. Reading the newspaper, I do standing as well, I don’t sit anymore, but keep standing.”* ***[P-NL3-12MFGD]***  *And also before ... at least I never stood up [at work]. I have a sort of adjustable desk, and now I stand approximately half of the day. It is up and [sit] down half of the day* ***[P-NOR2-PPFGD]***  *“It is not much but I try to get up and get some water from time to time in order to not stay sitting for long periods.”* ***[P-POR3-12MFGD]*** |
| **Eat a healthier diet** | *“Like I say, I ate well, but I'm conscious, I'm eating less, I'm having, my portion sizes are less. And I'm more, we don't, you know, we don't sort of sit and snack in the evenings, or if we do, it's fruit. And it's just things like, general things like that, you know.”* ***[P-UK4-PPFGD]***  *“Now I am aware what I can eat. I am more conscious.”* ***[5-NL3-PPFGD]***  *“I have changed my diet completely. It is totally radical; it is impossible to compare. Before I bought a lot of candy, like hot-dogs and Statoil-buns and that sort without thinking. Now that has totally stopped. So, when it comes to the diet, then it cannot be compared”* ***[P-NOR2-PPFGD]***  *“The more pronounced change was in my eating habits, I´ve changed pretty much everything… I´ve started to eat smaller quantities, choosing better, looking at the labels, eating more often, including breakfast…”* ***[P-POR3-PPFGD]*** |
| **Weight loss, waist reduction and reduced blood pressure** | *I: So, you said in the bookies the guy said you’d lost weight?*  *P7: Oh, you’ve lost weight. I says, yeah, I’m…*  *I: Yeah.*  *P7: …at EuroFIT…* ***[P-UK3-PPFGD]***  *“It is a tool. Look, if you are eating more conscious you will lose weight and become fitter. This will result in fewer problems during physical activity. If you are conscious about eating in the beginning, then this may result in a two-kilo weight loss. You don’t have to carry that two kilo during exercising. Exercising will go easier, which is motivating. You know, because it is easier, you are able to do more. This will result in acceleration; you are able to do more and more. I mean, I experience this myself. I can exercise more compared to the beginning. It is just because I lost seven kilos.”* ***[P-NL2-PPFGD]***  *“What I thought all the time really that it was very fantastic that you focussed on walking, and I did lose my 15 first kilo on only walking. I did not run a metre. And that, actually I was, in a way [I] knew that it was supposed to be possible, but that it was actually as effective as it was, that was totally crazy.”* ***[P-NOR1-310-PPFGD]***  “*Without any effort, only with very small changes I already lost 8 kg”* ***[P-POR1-PPFGD]*** |
| ***Longer Term Outcomes*** |  |
| **Influence others** | *P4: Umm, I…what we’re doing at home, we’re going out every night walking.*  *I: Yeah.*  *P4: Sometimes before tea, sometimes after tea, depends whether we’ve had the grandkids or not, you know, it’s every…every, err, night is different, but we do go out on a twenty-five-minute walk…*  *I: You and your wife?*  *P4: …yeah, every night up the…now up the canal as well…* ***[P-UK3-PPFGD]***  *“It is also a consciousness process for your children. If you are having diner and you are not taking it yourself then it is easier to say to your children not to take it either.”* ***[P-NL1-12M FGD]***  *When this project was ongoing, then I was at a department office that we, the bank where we work, we were three employees. So, they followed me quite closely then, in that project of mine. And I thought that they probably got tired [of it]. But then this fall now, then I moved back to the main office and then after the summer holiday and stuff, then I did not meet one of my colleagues there until close to Christmas, a big and nice man. Husky simply well-built [laughter]. Then it turned out that he, then he had suddenly lost 15 kilos that fall on the back of what I had been doing, on the back of that I had started with "knekkebrød" (high grain biscuits) for lunch, so he suddenly started with the same. So that was kind of fun to see.* ***[P-NOR1-12MFGD]***  *“All the family suffered some contagion… my wife started to eat breakfast and now goes for a walk with me (…) my daughter also used the SitFIT, we now compete with each other in the number of daily steps”* ***[P-POR3-12MFGD]*** |
| **Integration of new physical activity behaviours** | *I think the step thing has been really, really useful, and it’s almost second nature, when I get up in the morning, where’s the step thing, put it on straightaway, and the odd occasion when I forget it, I feel naked without it, so I shouldn’t be doing anything, but I found the step thing really, really useful.* ***[P-UK2-12MFGD]***  *‘On your bike to the hockey fields. I already used to go to [name of club] by foot, but yeah that is five hundred meters so that’s not enough. But doing things like that structurally and three times a week I walk just for myself. And I do keep track of those.’* ***[P-NL2-12MFGD]***  *P5: Yes, or I feel that where I am now, and then I see ... I feel like I have cracked the code.  I: Yes.  P5: Because that is exactly what I have not done in 20 years, not to overestimate. This arrangement has cracked the code. And then ... And then it is not so hard anymore.* ***[NOR2-12MFGD]***  *“I discovered cycling and other gym activities; I often go 5 times a week and run at weekends!”* ***[P-POR3-12MFGD]*** |
| **Better long-term health outcomes** | *P1: As a mental health nurse, I think yes, improve our mental health also, you do exercise, you think well.*  *I: Okay, so you sometimes feel better in yourself.*  *P1: Mentally healthy, yeah.* ***[UK1-12MFGD]***  *Yeah, the program was simply a boost because you got a boost to keep it going, it is as it has been said around the table here, that it ... some of us have managed to keep it up (noise). If the level is not 100 % where we want, then we are at least much, much, much more active then we used to be. Your [physical] form is better, and some have lost some kilos and the workday is easier.* ***[P-NOR-12MFGD]***  *“I´ve lost 25 kg… some time ago I picked a bag with 25 kg, and I was surprised… How could I carry such an amount of weight for so long?”* ***[P-POR1-12MFGD]*** |
